# Copper Requirement and Acquisition by Marine Microalgae

**DOI:** 10.3390/microorganisms10091853

**Published:** 2022-09-16

**Authors:** Liangliang Kong

**Affiliations:** College of Marine Life Sciences, Ocean University of China, Qingdao 266003, China; liangliang.kong@mail.mcgill.ca

**Keywords:** copper, Cu requirement, Cu uptake, Cu reduction, marine microalgae

## Abstract

Copper is a critical metal nutrient required by marine microalgae but may be toxic when supplied in excess. Maintaining an optimal intracellular Cu content is thus fundamentally necessary for microalgae and relies on cellular regulatory metabolisms and the process of Cu uptake that buffers the variation in environmental Cu availability. In this article the current progress in understanding the Cu requirements and acquisition mechanisms of marine microalgae is reviewed. Cu requirement by microalgae is primarily determined by the amount of Cu-dependent enzymes involved in cellular metabolisms and can be adjusted by Cu-sparing pathways. Decrease in metabolic Cu quotas caused a decline in the abundance of cuproenzymes and the dependent cellular metabolisms, and an induction of Cu acquisition pathways. Conventional models of Cu uptake describe the dependence of Cu uptake rate on free Cu^2+^ ions or kinetically labile species. A reductive, high-affinity Cu uptake system in marine microalgae is identified which enables cells to directly utilize organically complexed Cu, highlighting the importance of cell surface Cu reduction in the marine Cu cycle. This review provides new insights into Cu uptake models that may update the existing knowledge of Cu availability in the ocean.

## 1. Introduction

Marine microalgae require both macronutrients (e.g., nitrogen, phosphorus, silica) and micronutrients (e.g., vitamin and trace metals) to grow. The trace metals mainly function in metabolic pathways as cofactors of enzymes, and their availability is a crucial environmental factor regulating algal growth [1,2]. Copper (Cu), one of about ten known essential metals, is indispensable for microalgae because of its role as a cofactor of redox proteins involved in electron transfer and oxygen cycling [3,4]. Meanwhile, free intracellular Cu is toxic to cells because it produces harmful reactive oxygen species and disrupts protein function by displacing other metal cofactors from critical sites [5,6,7]. In the ocean, dissolved Cu concentrations in surface waters are so low [8,9,10] that the supply of Cu may be less than its demand by many microalgae, leading to Cu limitation in some remote regions of open seas [11,12], while in some Cu-enriched coastal regions, elevated Cu levels may have negative effects [13,14]. Acquisition of Cu by marine microalgae is thus of fundamental importance because it controls the entry of Cu into cellular storage pools and buffers the variation in ambient Cu availability [15]. Conventional models of metal uptake by marine microalgae describe the dependence of uptake on the concentration of hydrated metal ions or kinetically labile species [16]. Some early studies on Cu toxicity show that biological responses of marine microalgae to Cu were directly related to the concentration of free cupric ion but not the total Cu [17,18,19]. More recent experimental and modeling results suggest organically complexed Cu, the predominant forms in the dissolved Cu pool in surface seawater, is also accessible to marine microalgae [20,21]. Experimental evidence for direct utilization of organic Cu by marine diatoms was recently explored and established, highlighting the critical role of organic forms of Cu and the reductive Cu uptake pathways in the marine Cu cycle [22,23]. Here, we review the current progress in Cu requirements and acquisition mechanisms of marine microalgae. The characterized reductive Cu uptake pathways enable marine microalgae to directly utilize organically complexed Cu; these findings may update the existing knowledge of Cu availability in the ocean.

## 2. Copper in the Ocean

### 2.1. Cu Concentration and Distribution

Dissolved Cu, like other essential trace metal nutrients, is present at very low concentrations in surface oceans due to the low solubility of Cu in alkaline seawater (e.g., Cu(OH)_2_) and biological uptake [1,24,25]. Total dissolved Cu concentration varies between ~0.5 to 3 nM in offshore, surface seawater and increases from lower to higher latitudes and in coastal regions [8,9,10,20,25,26,27]. The elevated Cu concentration in coastal waters, up to 50 nM, is a result of a number of processes, including upwelling of Cu-rich deep water, fluvial and aeolian input, and anthropogenic pollution, and may inhibit algal growth [13,28,29,30]. Vertical profiles of dissolved Cu in the ocean show a nutrient-type distribution with low concentration in the sea surface and high concentration at depth [9,24,31]. Remineralization of sinking particles and diffusion of dissolved Cu from ocean sediments contribute to the high concentration in deep waters. Unlike a typical nutrient profile that shows a rapid increase below nutricline, dissolved Cu concentration increases linearly with depth, possibly due to remineralization and reversible scavenging of Cu in deep waters [20,24,32].

### 2.2. Cu Chemical Speciation

Chemical speciation of Cu in the ocean is controlled by complexation reactions with organic ligands that bind more than 99.7% of the total dissolved pool [8,31]. Two classes of organic ligands are identified according to their Cu-binding affinity: a strong ligand class (L_1_) present at relatively low concentrations of 2–4 nM with conditional stability constants log K′ = 13–16, and a weak ligand class (L_2_) present at relatively high concentrations of 5–10 nM with log K′ = 8–13 [10,33,34,35]. Distribution of L_1_ ligands tightly correlates with Cu but with a slightly higher concentration in the upper ocean [10,35]. Elevated L_1_ in surface waters has been interpreted to imply a biological origin of the ligand. Indeed, marine cyanobacteria are known to secrete strong Cu-binding ligands that are comparable to the natural L_1_ ligands in response to Cu exposure [34,36]. So far, the composition of natural Cu ligands has not been elucidated, but they appear to contain some thiols, humic substances and chalkophores [35]. Strong complexation by L_1_ reduces the equilibrium concentration of Cu^2+^ to femptomolar levels (10^−15^–10^−14^ M) [9,10,33,35].

Copper also exists in the ocean in two oxidation states, Cu(I) and Cu(II). Cu(II) is thermodynamically favored in oxygenated waters, but some Cu(I) may also be present. Current estimates are that about 10% of the total dissolved Cu in surface waters exists as Cu(I), stabilized by chloride ions or thiol ligands [37,38,39]. Photochemical processes that lead to ligand to metal charge transfer [39,40], and superoxide-mediated reduction [41], are thought to contribute to the bulk of Cu(I) production in the ocean. Biologically mediated Cu(II) reduction may also be important [23,42,43].

### 2.3. Cu Bioavailability

Metal availability and speciation regulate microalgae community composition and growth and thus affect ocean primary production [2,44]. Boyle and Edmond [45] proposed that Cu was a limiting resource in surface waters of the southwest Pacific Ocean based on their observation that the dissolved Cu:P ratio in seawater was less than in marine algae. Growth of diatoms in laboratory cultures is limited at Cu concentrations of 10^−15.1^–10^−14.4^ M free Cu^2+^, similar to levels found in the open sea [12,22,46]. Thus, Cu may naturally limit growth of diatoms in parts of the ocean. Indeed, addition of 2–4 nM Cu significantly stimulated phytoplankton growth and production rates in the subarctic Pacific Ocean [11,12], confirming that Cu is a limiting resource. Copper deficiency in marine microalgae may be more severe in low Fe environments, such as the high-nutrient, low-chlorophyll regions, because the high-affinity Fe uptake system contains a Cu-dependent ferroxidase and its up-regulation requires more Cu [12,47,48].

Copper toxicity occasionally occurs in some Cu-enriched environments, such as coastal regions that may receive a large quantity of atmospheric aerosols containing high Cu concentrations and is thought to alter the abundance of particular microalgae [13,30,49,50,51,52]. Because of the difference in metabolic Cu requirements and capacity of Cu uptake in different species, marine microalgae show interspecies differences in Cu sensitivity, among which cyanobacteria are the most sensitive microalgae to Cu toxicity (reproduction inhibited by [Cu^2+^] above 10^−12^ M) and eukaryotic algae more tolerant to Cu overload, assessed by growth performance at high Cu concentrations [51,53,54,55]. To minimize its toxic effects, cyanobacteria secrete strong Cu-binding ligands that complex Cu and reduce the concentration of bioavailable Cu in the environment [36,56]. Some eukaryotic algae also secrete Cu ligands, but with weaker binding affinity, in response to high Cu stress [56,57]. Eukaryotes evolved a shuttle system that delivers Cu to specific targets via Cu chaperons and maintains free cellular Cu concentration at undetectable levels [58]. Moreover, eukaryotic algae, for example, marine diatoms, contain an inducible Cu uptake system that tightly regulates Cu uptake and maintains optimal levels of cellular Cu [59].

## 3. Cu Requirement of Marine Microalgae

### 3.1. Cu Quotas

Metabolic Cu quotas are the amount of cellular Cu required by Cu-dependent metabolisms. The quotas are usually maintained within narrow limits by tightly regulated cellular processes even as environmental concentrations vary by orders of magnitude [59,60]. At low limiting concentrations, quotas invariably decline and become growth limiting, whereas at high concentrations, cellular Cu may accumulate to toxic levels. Cellular quotas are regulated by efficient Cu uptake and efflux systems to maintain an optimal storage of intracellular Cu that will ultimately influence growth rate of microalgae [59,61]. An oceanic diatom was able to grow in culture medium containing inorganic Cu (Cu′) concentrations varying by six orders of magnitude, primarily due to the presence of a tightly regulated, high-affinity Cu uptake system [22].

Cu quotas also vary among species by a factor of 10 [62] because of differences in cellular metabolisms. For example, oceanic diatoms require 10-fold more Cu for growth than closely related coastal isolates, primarily due to the presence of a Cu-containing protein, plastocyanin, that is absent in other coastal species [63]. It is estimated that about 50–70% of intracellular metabolic Cu is allocated to plastocyanin in diatoms and green algae [46,63,64]. Copper also plays an essential role in iron nutrition because Cu is required for high-affinity Fe uptake [65]. Cellular Fe quotas and uptake rates decrease in Cu-limited diatoms [12,66] and more Cu is required for growth when Fe is limiting [66]. Field observations had shown cellular stoichiometric Cu:P ratios were higher in microalgae in the Southern Ocean, where Fe limitation prevails [67,68], than in the Equatorial Pacific Ocean and the North Atlantic Ocean [69], indicating a higher Cu requirement for microalgae in low Fe environments [20,32]. A decrease in environmental Cu concentration reduces the cellular Cu:C ratio in microalgae [59,66] and presumably Cu-dependent pathways are down-regulated. Growth rate declines coincidentally and is positively correlated to cellular Cu content under Cu-limiting conditions [46].

### 3.2. Cuproenzymes

The rise of dissolved oxygen produced by oxygenic photosynthesis changed the redox state of the paleoocean, greatly increasing the solubility of dissolved Cu and decreasing the bioavailability of iron [70]. Since then, Cu has been available to organisms and utilized as a cofactor of high redox potential in metalloenzymes [71]. Except for a few Cu-dependent proteins (cuproenzymes) in aerobic bacteria and cyanobacteria, most cuproenzymes are found in eukaryotes; these organisms evolved after the increase in Cu availability in oxygenated seawater [70,72,73]. The cuproenzymes of eukaryotes primarily function in redox reactions involved in electron transport and oxygen chemistry [3,4].

The three most abundant cuproenzymes in eukaryotic microalgae are plastocyanin, cytochrome *c* oxidase and multicopper oxidase [64,74]. Plastocyanin is a photosynthetic electron transfer protein that transfers electrons from cytochrome b_6_/f complex to photosystem I [75]. Cytochrome *c* oxidase catalyzes the final step of respiration by reducing oxygen to water [76]. Multicopper oxidase is a Cu-containing ferroxidase and functions in iron acquisition [48,65]. Other important cuproenzymes in algae include amine oxidase involved in amino acid metabolisms, Cu-Zn superoxide dismutase for free radical scavenging, urate oxidase in the urea cycle, tyrosinase and laccase for betalain and melanin synthesis [77,78,79,80]. Some low molecular weight proteins found in algal cells also bind Cu, including metallothioneins and phytochelatins, but are not considered as cuproenzymes, because they bind Cu temporarily as a detoxification strategy and Cu does not regulate their functions as a cofactor [81,82].

### 3.3. Cu-Regulated Metabolisms

Under Cu deficiency, the cellular concentration of cuproenzymes declines with the decrease in the amount of cellular Cu [64]. Plastocyanin, for example, the most abundant cuproenzyme in photoautotrophs, is down-regulated by more than 3-fold in Cu-limited diatoms [83,84] and decreased to an undetectable level in Cu-limited *Chlamydomonas* [74,85]. Down-regulation of plastocyanin allows Cu to be relocated to cytochrome *c* oxidase and multicopper oxidase [86] and the loss of plastocyanin function in *Chlamydomonas* can be restored by up-regulation of a Fe-containing protein, cytochrome *c*_6_ [87]. The replacement of cuproenzymes with functional equivalents is also shown in Cu-containing amine oxidase and multicopper reductase, which can be substituted by a flavin-dependent amine oxidase [74] and a CRD2 protein [47,88] under Cu limiting conditions. If such Cu sparing mechanisms are absent in microalgae—for example, oceanic diatoms only contain plastocyanin but no functional cytochrome *c*_6_ [84], then a decrease in the abundance of cuproenzymes will cause defects in cellular metabolisms and reduce growth rate. For example, Cu deficiency caused downregulation of most anabolic metabolisms and energy-yielding reactions, such as photosynthesis, carbon fixation, nitrogen assimilation and glycolysis, to accommodate slower growth of a plastocyanin-dependent diatom [84,89]. Because of the critical role of plastocyanin in photosynthesis and its binding of the majority of Cu quotas, the initial targets of Cu deficiency are believed to be localized in chloroplasts of plastocyanin-containing species where decreased abundance of plastocyanin impaired photosynthetic electron flow and oxidative defense systems were activated primarily in chloroplasts to counterbalance low Cu-induced oxidative stress [89].

## 4. Cu Acquisition Mechanisms

### 4.1. Cu Uptake and Cu Transporters

Copper uptake is of great importance in maintaining cellular Cu homeostasis, because it controls the amount of Cu that is transported into cells. Uptake proceeds by a two-step reaction involving binding of the metal to a specific transport ligand on the cell surface followed by internalization [90]. In marine and freshwater microalgae, the Cu uptake rate follows a saturation curve, described by Michaelis–Menten kinetics [91,92,93]. In some species, uptake is biphasic and includes both a low and a high-affinity Cu uptake system [93,94]. Half-saturation constants (Km) of the low-affinity uptake systems are between 1.82 to 15.5 pM free Cu^2+^ (pCu = 10.8 to 11.7; pCu = −log[Cu^2+^]) in marine cyanobacteria and diatoms [91,93,94] and thus thought to function at high environmental Cu concentration. High-affinity Cu uptake was first described in yeast and selectively transports monovalent Cu ions (Cu^+^) via specific cuprous transporters [95,96]. In the green alga, *Chlamydomonas*, the high-affinity system is induced by low Cu and increases the uptake rate by 20-fold in Cu-limited compared to Cu-replete cells [92]. Half-saturation constants of the high-affinity uptake systems in freshwater and marine algae are between 5.62 and 68.4 fM Cu^2+^ (pCu = 13.2 to 14.2), more than 100-fold lower than the Km of low-affinity uptake systems [93,94]. Thus, high-affinity Cu uptake is considered to be more important for marine microalgae in low Cu environments where effective Cu scavenging is at a premium.

High-affinity Cu uptake systems feature high specificity for Cu^+^ transportation and consist of two molecular components, high-affinity CTR-type Cu transporters (CTR) and cupric reductases [97]. CTRs were first described in yeast [95] and later found to be widespread in eukaryotes. CTR proteins contain essential amino acid motifs and protein domains that are conserved throughout eukaryotic lineages [60,97]. The conserved regions include methionine and/or histidine-rich motifs in the N-terminus for capturing Cu at the cell surface, an ion channel constituted by three α-helices in the transmembrane domains for transporting Cu, and cysteine and/or histidine motifs in the C-terminus for delivering Cu to intracellular chaperones. An amino acid motif MxxxM-x_12_-GxxxG in transmembrane domains is characterized as the signature motif found in all CTR proteins [98]. The N-terminal methionine motifs preferably bind Cu(I) [99], and Cu uptake via CTR transporters is facilitated by Cu(I) but not Cu(II) in yeast [96]. CTRs are also present in vacuolar membranes of yeast and plant cells, shuttling Cu between vacuole and cytoplasm but with a relatively low affinity [100,101,102]. Marine species also contain homologues of CTR transporters and their function in Cu transportation was examined in an oceanic diatom, *Thalassiosira oceanica,* by functional complementation of a CTR-defect yeast mutant [22]. Expression of CTR genes of *T. oceanica* in the yeast mutant successfully restored growth in Cu-deficient medium and the expressed proteins selectively transported Cu(I). A closely related coastal isolate, *T. pseudonana*, also contained candidate CTR genes, but the expressed proteins failed to restore growth of the yeast mutant [103]. The possible reason could be that coastal species have a lower Cu requirement and thus do not need a high-affinity uptake system to acquire Cu in coastal regions containing relatively high Cu concentrations. Gene expression of CTRs is regulated at transcriptional level by cellular Cu nutritional state and inducible by environmental concentrations [60]. For example, CTR is highly expressed in Cu-limited algal cells [22,60,74] and degraded in response to high Cu exposure [104,105].

Low-affinity uptake proceeds by some non-specific divalent metal ion transporters. One type of this assimilatory Cu transporter is the NRAMP (Natural Resistance-Associated Macrophage Protein) family of divalent metal transporters. NRAMP was named for its first detection in membranes of macrophages and its function in the digestion of intracellular parasites [106]. The human NRAMP (NRAMP1, NRAMP2) and its homologues in yeasts (SMF1, SMF2, SMF3) and mammals (DMT1/DCT1) are general metal ion transporters that transport a broad range of metal substrates across the plasma membrane, including Fe^2+^, Mn^2+^, Co^2+^, Zn^2+^, Cd^2+^, Cu^2+^, Ni^2+^ and Pb^2+^ [107,108]. The NRAMP family proteins have ten to twelve conserved transmembrane domains, but their function in metal transport as a proton/metal ion symporter or an antiporter remains controversial [108,109]. The marine diatom, *T. pseudonana*, contains an NRAMP homologue protein (TpNRAMP) in its genome and its expression increases by 2-fold in Cu-limited cells [110]. Very similar to NRAMP, the ZIP (Zrt, Irt-like Protein) family of metal transporters that were originally identified as zinc and iron transporters in yeast and *Arabidopsis* [111,112,113] use a broad range of substrates [114]. Inhibition of Zn uptake by Cu in *Arabidopsis,* suggested that ZIP may also transport Cu [115]. Expression of one ZIP gene is upregulated by 20-fold in Cu-limited *Chlamydomonas* [74], but the molecular mechanism of ZIP-mediated Cu uptake in marine species is still unknown.

### 4.2. Cu Reduction and Cupric Reductases

The first description of Cu reduction in marine algae showed that Cu(II) bound to inorganic and organic complexes was reduced to Cu(I) extracellularly [42,116]. Extracellular Cu(II) reduction was later observed in other species, including diatoms, green algae and in a coccolithophorid [23,92,117,118,119]. The importance of redox cycling of Cu in the ocean was initially considered in a chemical context, as a catalyst in photochemistry of carbon and oxygen cycling in the ocean [39]. Not until high-affinity CTR cuprous transporters were characterized in yeast [95], was the biological function of Cu reduction appreciated as an important step in Cu uptake. Inhibition of Cu reductase activity in yeast reduced the Cu uptake rate substantially, providing the first experimental evidence for the dependence of Cu reduction on uptake [96]. The presence of functional CTRs in marine diatoms implied that extracellular Cu(II) reduction was a necessary step in Cu uptake, because dissolved Cu is present predominantly as oxidation state of Cu(II) in the surface ocean [38]. Addition of bathocuproinedisulfonate, a strong Cu(I)-binding ligand, decreased Cu uptake rate of a marine diatom by more than 75% and completely inhibited growth in Cu-deficient medium, demonstrating Cu(II) reduction was an obligatory first step in Cu uptake in this species [23].

The molecular mechanism of cell surface Cu reduction in marine species is poorly understood, but there is some evidence that it is associated with nitrate reductase activity [42,116]. In yeast, cell surface Cu reduction is mediated by two ferrireductases, FRE1 and FRE2 [96,120], that were initially described for Fe reduction [121,122]. FREs belong to the protein superfamily of flavocytochromes and are homologous to the β-subunit, gp91^phox^, of human NADPH oxidases [123]. NADPH oxidase and its protein homologue, the respiratory burst oxidase in plants, catalyze the production of superoxide radicals by reducing oxygen with electrons donated from intracellular NADPH and transferred by two cofactors, flavin adenine dinucleotide (FAD) and two b-type hemes [124,125]. Putative FREs identified in marine diatoms (e.g., *Tp*FRE1,2 of *T. pseudonana*, *Pt*FRE1,2 of *Phaeodactylum tricornutum*, *To*FRE1,2 of *T. oceanica*) share conserved amino acid motifs and protein domains with yeast FREs and NADPH oxidases [43,126,127,128]. These include multiple transmembrane domains, FAD and NADPH binding motifs on the cytosolic side, and a bis-heme motif in transmembrane domains.

Biochemical function of cupric reductases was originally investigated in *Saccharomyces cerevisiae* [96,120]. A small number of studies had examined the identity and function of cupric reductases in marine microalgae; they showed that expression of putative FRE genes is regulated by Cu in marine diatoms, indicating a possible role in Cu homeostasis [89,110]. Cupric reductase activity of two FRE genes of a marine diatom were recently validated by functionally complementing a Cu reduction-deficient yeast mutant. Transformation and expression of diatom FRE genes in the yeast mutant significantly increased the rate of Cu reduction by more than 2-fold relative to the non-transformed mutant strain [43]. Some studies suggest metal ions may be directly reduced at the enzymatic site of reductases or via some intermediate metabolites, such as superoxide anions (O_2_^−^) [128,129], so the biochemical mechanism of how cupric reductases reduce Cu at the cell surface is still uncertain. Half-saturation constants of Cu reduction kinetics measured in marine microalgae are between 2 to 123 μM [42,117]. Given that the ambient Cu concentration is more than 1000-fold lower than measured half-saturation constants, enzymes that mediate Cu reduction may not function primarily for trace metal reduction and may have a more broad range of substrates of higher concentrations, such as nitrate and dissolved oxygen. In addition to biologically mediated Cu reduction, abiotic reduction of Cu occurs by photochemical processes and by superoxide [41,130], and may supply Cu(I) to marine algae.

### 4.3. New Insights into Metal Uptake Models

The initial work on the relationship between Cu availability and algae growth showed that the toxicity of Cu in freshwater and marine species was related to the concentration of free Cu^2+^ but not organically complexed Cu [17,19,53]. The general principles of the conventional metal uptake model, the free-ion activity model (FIAM), describe the dependence of growth and metal uptake on free metal ions or kinetically labile species and were applicable to many metals (e.g., Cd^2+^, Cu^2+^, Fe^3+^, Mn^2+^, Zn^2+^) [17,131,132,133]. Exceptions to FIAM were reported in more recent studies showing free Cu^2+^ or inorganic Cu′ species cannot fulfill the Cu requirements for uptake at growth limiting concentrations of Cu because of its extremely low concentration [66,134,135]. For example, rates of Cu uptake are 2 to 1000-fold faster than the rates of diffusion of Cu′ to the cell surface [21,66,134,135,136], so other chemical species of Cu are thought to be utilized directly by microalgae for growth. In EDTA-buffered growth media, rates of Cu reduction in a marine diatom depended on the concentrations of Cu(II)EDTA but not Cu′, further proving direct utilization of organic Cu by some marine algae [23]. Similar observations were shown for Fe and Zn uptake when measured rates of metal uptake exceeded the maximum diffusion flux rate of inorganic species to the cell surface [137,138]. It has been shown that marine microalgae are able to utilize Fe bound to siderophores, and extracellular reduction of organic Fe makes Fe available to algal cells [138,139,140]. Alternatively, siderophore-bound Fe could be transported directly into cells as a complex by endocytosis-mediated siderophore uptake [141]. Acquisition of Zn could be facilitated with weak binding ligands and transported as a Zn-cysteine complex through cysteine specific transporters [137]. So far, direct transport of organic Cu complexes has not been reported. Instead, the reduction-dependent, high-affinity Cu uptake system characterized in marine diatoms enables cells to utilize organically complexed Cu after it is reduced at the cell surface [22,23].

**Figure 1:**
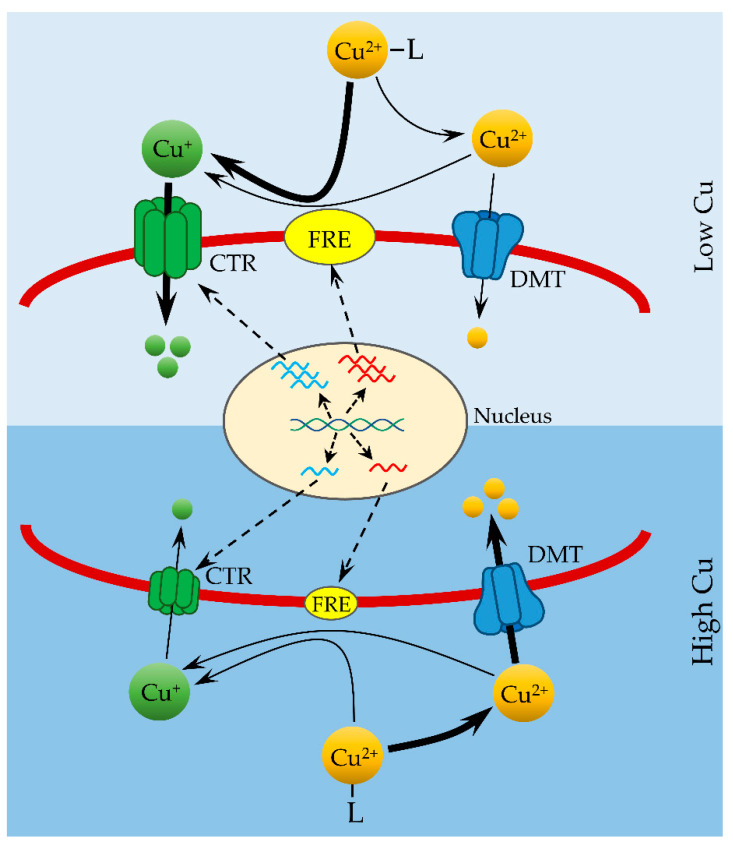
Model of copper uptake by a marine diatom in low and high Cu environments. The graph was replotted according to data reported by Kong and Price [22,23,43,89]. Organically complexed Cu (Cu-L) is dissociated to Cu^2+^ by equilibrium or to Cu^+^ by reduction at the cell surface. Cu^+^ is transported across the cell membrane by high-affinity CTR cuprous transporters and Cu^2+^ is transported into cells by low-affinity divalent metal transporters (DMT). Expression of CTR transporters and reductases (FRE) are regulated by environmental Cu concentrations. A larger size of the CTR and FRE symbols represents higher abundance of expressed proteins in low Cu environments. The dominant pathway at low or high Cu is identified by the thick lines.

The possession of both high-affinity (reductive) and low-affinity (nonreductive) Cu uptake systems allows marine diatoms to adjust strategies to acquire Cu from different environments (e.g., low Cu and high Cu environments). For example, at growth-limiting, low Cu environments, concentrations of Cu′ (including free Cu^2+^) are so low that uptake of Cu′ via low-affinity transporters contributes only a negligible amount to the bulk uptake of Cu from the environment (Figure 1). Instead, molecular components of high-affinity Cu uptake system, including CTRs and FREs, are highly expressed in response to low Cu (Figure 1). An upregulated high-affinity uptake system reduces organic Cu (Cu-L) at cell surface and makes it available for transport via CTRs. Because concentrations of Cu-L are more than 1000-fold higher than Cu′ in strong ligand-buffered (e.g., EDTA) culture medium and natural seawater, the reductive Cu uptake pathway predominates in the process of Cu uptake and relies on total Cu concentration under Cu limiting conditions. At high Cu environments, the reductive pathways are downregulated and concentrations of Cu′ are high enough to meet the requirements of Cu uptake by marine microalgae (Figure 1). Addition of a strong Cu(I)-binding ligand did not affect algal growth at high Cu concentrations [23], indicating that Cu is transported as the oxidation state of Cu(II) by the low-affinity uptake system and that the nonreductive pathway predominates in the bulk uptake of Cu at high Cu environments (Figure 1). Thus, the principles of FIAM are still valid but only at high environmental Cu concentrations. The presence of reductive Cu uptake pathways in some marine species allows the utilization of organic Cu and its regulation by environmental Cu availability provides new insights into metal uptake model and highlights the importance of organic Cu in marine Cu cycles.

## 5. Conclusions and Future Perspectives

Marine microalgae adjust cellular metabolisms and regulate Cu uptake to maintain an optimal intracellular Cu content and growth when growing in environments with variations in environmental Cu availability. Cu deficiency causes a decrease in metabolic Cu quotas, which reduces the abundance of cuproenzymes and affects the dependent cellular metabolisms. Cu uptake is inducible in response to Cu availability and its regulation by cellular Cu nutritional state buffers the changes in environmental Cu concentrations. The possession of a reductive Cu uptake pathway allows marine diatoms to directly utilize organically complexed Cu, the previously unrecognized forms of bioavailable Cu. CTR transporters are widespread in eukaryotes that include marine microalgae so that other marine species have the potential to acquire Cu by the same CTR-mediated Cu pathway, especially in open oceans where Cu is predominantly complexed by strong ligands and present at extremely low Cu concentrations. The high-affinity Cu uptake pathway requires a prerequisite Cu reduction step that Cu(II) is reduced to Cu(I) prior to Cu internalization. Biological and photochemical reactions that catalyze reactions of Cu(II) reduction are thus fundamental to Cu uptake by marine microalgae in the ocean. Current views recognize that extracellular Cu(II) reduction is required for Cu(I) uptake by Cu(I) transporters, but pay little attention to the reduction step [54,93,94]. A recent study shows Cu(II) reduction is the rate-determining step in Cu acquisition by a diatom and identifies a previously overlooked regulatory step in Cu uptake [43]. If the reduction step determines the rate of Cu uptake and the reductive pathways are widespread in marine microorganisms, then the properties of the Cu complexing ligands and the chemical or biological reactions for redox cycling of Cu should be considered primarily, rather than the chemical equilibrium of inorganic Cu species, when determining Cu availability in the sea. This is relevant to understanding how environmental (e.g., Cu concentrations, light, temperature) and chemical (e.g., electrostatic and steric factors, and reduction potentials of organic Cu complexes) parameters affect Cu uptake by marine microalgae in the sea and biogeochemical cycling of metals in aquatic ecosystems.

## Data Availability

Data supporting this study are available in cited articles where they were originally reported.
